# The Application of Laser Acupuncture in Animal Experiments: A Narrative Review of Biological Aspects

**DOI:** 10.1155/2021/6646237

**Published:** 2021-02-24

**Authors:** Yan Yang, Gerhard Litscher, Zhongren Sun, Wen Sun

**Affiliations:** ^1^Research Unit for Complementary and Integrative Laser Medicine, Research Unit of Biomedical Engineering in Anesthesia and Intensive Care Medicine, and TCM Research Center Graz, Medical University of Graz, Auenbruggerplatz 39, Graz 8036, Austria; ^2^Heilongjiang University of Chinese Medicine, Harbin 150040, China; ^3^Heilongjiang Mental Hospital, Harbin 150036, China

## Abstract

Experimental studies on animals are conducted in almost all areas of medical research. The experiments offer insights into diseases and expand biomedical knowledge. Animal experiments are also imperative for studying acupuncture treatment mechanisms and the exploration of innovative acupuncture techniques. Laser acupuncture (LA), as a promising alternative to traditional manual acupuncture (MA), has the characteristics of painless and controllable stimulation. Today, society is more aware of animal welfare than previous generations. The use of noninvasive LA as a substitute for invasive MA in basic experimental research of acupuncture should be encouraged. Thus, we conducted an overview of animal experiments in the research field of LA from January 1978 to April 2020. After careful research, 52 studies were included in the work. Among these studies, both single-point and multipoint LA studies have been reported. To make noninvasive LA better in replacing invasive MA in experimental animal research of acupuncture, further experiments should focus on exploring uniform criteria for selecting laser parameters and revealing the extent to which the curative effect of LA depends on the property of acupuncture points rather than the characteristics of photobiomodulation.

## 1. Introduction

Laser acupuncture (LA) has been defined as “*photonic stimulation of acupuncture points and areas to initiate therapeutic effects similar to that of needle acupuncture and related therapies together with the benefits of photobiomodulation (PBM)*” [1]. A variety of lasers, such as solid-state lasers, semiconductor lasers, gas lasers, or dye laser, can be used in LA treatment [2]. With noninvasive and painless properties, LA is becoming more and more accepted, especially among children and patients with needle phobia. A recent review article indicated that laser acupuncture is a relatively safe therapy with few mild and transient adverse events [3]. Although LA has first been used clinically in the 1970s [4], in just 50 years of development, the research of LA has reached a global scale [5]. Evidence was found to support the use of LA in the treatment of myofascial pain [6], postoperative nausea and vomiting [7], chronic tension headaches [8], musculoskeletal pain [9], and asthma [10]. Technical parameters such as wavelength and intensity are crucial in LA treatment. By adjusting laser parameters, equivalence between metal needles and laser needles can be achieved. LA also presents as a promising alternative method of manual acupuncture (MA) in study design. In terms of the optical laser needle stimulation, systematic, double-blind studies [11] can be carried out for the first time in an acupuncture study due to the patient ignorance of the activation or deactivation of the laser needles. As with other drug therapy studies, animal experiments are essential for the in-depth research of acupuncture mechanisms. At present, MA and electroacupuncture (EA) have been applied in many animal experiments. However, although the clinical use of LA is already widespread, due to its relatively recent origin and its clinical emphasis, there are comparatively few studies dealing with fundamental research on this topic. Compared to EA and MA, LA seems more suitable for a wide range of experimental animal research due to its painless and controllable stimulation. The animal experiments using LA are more likely to reflect the acupuncture treatment mechanism comprehensively and accurately and thus provide the best treatment plan for clinical acupuncture treatment of various diseases. Nevertheless, how the laser interacts with human and animal tissues and what other effects LA has beyond traditional acupuncture still need to be further studied [12].

In this article, we will review the animal experimental investigations concerning LA research to analyze the application status and development prospect of LA in basic research of acupuncture.

## 2. Search Strategy

**Figure 1:**
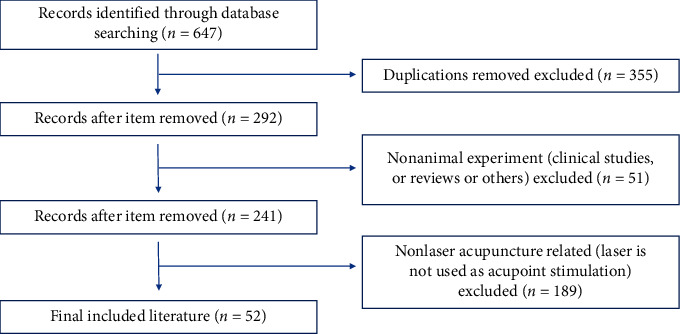
Flowchart of search strategy and selection of the literature.

We conducted an English-language search of databases, including PubMed, Web of Science, EMBASE, and Cochrane Library. Database searches were performed using the following terms: (acupuncture OR acupoint OR moxibustion) AND (laser) AND (animal OR rat OR mouse OR rabbit). Literature from January 1978 to April 2020 was included. Animal experiments were our main concern. By reading obtained references seriously, we present the final included citations in this review. The search strategy is shown in Figure 1.

647 records were identified. After careful research, 52 articles were found directly involved in the experimental animal research on LA. Among these publications, the choice of experimental animals is mostly rodents (rats and mice), followed by cats, rabbits, and gorillas. Both single-point and multipoint laser acupuncture studies have been reported. The research results show that LA has the effects of analgesia, anti-inflammatory aspects, regulating endocrine, promoting blood circulation, etc. However, the current animal experimental research of LA only involves a small number of acupoints. There is no uniform standard for the intensity of laser stimulation at acupoints as of yet.

## 3. LA on Single Point

Using lasers to explore the mechanism of action of a single acupuncture point or a combination of multiple acupuncture points has promoted acupuncture development. In TCM (traditional Chinese medicine) theory, each acupoint has its own characteristics according to the meridian to which it belongs and the position where it locates. The application of various acupoint stimulation methods may have different effects on acupoint function. Basic research of LA on single acupoint will help to improve the efficacy of LA in clinical practice.

### 3.1. ST-36

Zusanli (ST-36) is one of the most commonly used acupoints in clinical practice; according to TCM theory, ST-36, which is located at the stomach meridian on the lower leg, has a close relationship with gastrointestinal function. It is an essential point in health care, and it has been widely used in the prevention and treatment of various diseases. Acupuncture treatment at ST-36 has been frequently investigated in animal experiments. Laboratory studies have proved that ST-36 has the effect of regulating autonomic nerve activity [13], has further anti-inflammatory effects [14], and is enhancing the body's immunity [15]. Various types of LA methods have been used in experimental animal studies on ST-36. Invasive examination, including blood tests, indicated that LA at ST-36 induced changes in cellular and molecular levels, mostly affecting the mechanisms related to pain relief. The first animal experimental study of LA on ST-36 was published in 1989. Bian et al. [16] reported for the first time in China the effect of semiconductor GaAs (gallium arsenide) laser irradiation at ST-36 on the pain threshold of rabbits. Their results showed a noticeable analgesic effect, especially after 15 minutes of laser stimulation. The conduction of peripheral nerves and the release of endogenous opioid substances were suggested to be closely related to the analgesic effects of acupoint laser stimulation. Ten years later, another study [17] was conducted to explore the analgesic effects of LA on arthritic rats with single and low-intensity composite laser irradiation at ST-36. 650 nm single laser and compound 10.6 *μ*m + 650 nm laser were found to increase the pain threshold of rats. Mast cell degranulation rate was believed to play an important role in laser irradiation-induced analgesia by being positively correlated with analgesic effects. Erthal et al. [18] evaluated the effect of LA on reducing pain-related behavior in rats. An antinociceptive effect of LA at ST-36 was found in an acute noxious rat model, and opioidergic and serotonergic (5-HT1 and 5-HT2A receptors) systems were involved in producing this effect. Two years later, the same research group [19] demonstrated that low-level laser (LLL) pretreatment on ST-36 could inhibit the mechanical allodynia (von Frey) response induced by partial sciatic nerve ligation (PSNL) and Complete Freund's Adjuvant (CFA). Besides, they also suggested [20] LLL treatment could reduce edema, temperature, and free radicals' levels in the mice model of carrageenan-induced paw edema via anti-inflammatory mechanisms. For acute pain control, Zeng et al. [21] found that repetitive low-level laser acupuncture (LLLA) reduced plantar incision-induced mechanical pain as much as EA did. Thus, they suggested LLLA can be used as an alternative to EA for postoperative pain control. LA pretreatment also has an inhibitory effect on visceral traction pain (VTP), 650 nm laser irradiation at ST-36 has been proved to alleviate VTP in rats, and the underlying mechanism may be related to decreased AChE activity and SP content, increased leu-enkephalin (LEK) activity, and downregulation of c-Fos protein and glial fibrillary acidic protein (GFAP) expression [22]. In addition to its analgesic effects, LA can affect local organ metabolism, and its effect could be associated with the function of acupoint and the biostimulative effect of the laser. Frederico et al. [23] indicated that laser irradiation could interfere with the metabolism of the thyroid, which caused increased absorption of radiopharmaceutical Na99mTcO4 in rats. To enhance the stimulating effect of LA, Zhang et al. [24] inserted an optical fiber needle into ST-36 of mice and observed blood changes with Raman spectroscopic technique. A low concentration of porphyrin, NADH, lipid, and glucose accompanied by a high concentration of phosphatidylinositol in blood was found after applying optical fiber acupuncture. Liu et al. [25] explored the role of LA on hypoxia tolerance and inflammation reaction in mice; they inserted optical fiber-fabricated acupuncture needles into ST-36 of mice for laser treatment. Improvement of tolerance to hypoxia and elevation of serum IL-1 level was observed after treatment.

### 3.2. PC-6

Neiguan (PC-6) is a point on the pericardium meridian which lies between the tendons of palmaris longus and flexor carpi radialis muscles, 2 *cun* above the wrist crease. Stimulating PC-6 is essential to treating some heart diseases, mental disorders, nausea, and vomiting [26, 27] caused by various reasons according to TCM theory. The molecular mechanism of acupuncture at PC-6 on myocardial ischemia [28] has been widely reported in animal experiments and the animal experiments which investigate the effect of LA at PC-6 mainly focus on its impact on heart rate (HR) and heart rate variability (HRV). Zhang et al. [29] preliminarily explored the effect of low-power helium-neon laser irradiation at PC-6 on the heart activity of cats by observing the changes of electrocardiogram (ECG). It was found that LA mainly affected HR. The HR of most cats slowed down after laser irradiation. Two decades later, another research group [30, 31] from China compared the effects of different LLLT on rabbits with bradycardia induced by injection of pituitrin. The result reflected that stimulation on PC-6 with 10.6 *μ*m CO_2_ laser significantly improved bradycardia while the 650 nm semiconductor laser does not produce such an effect. However, when the two lasers are combined, the effect is positive. Instead of focusing on the type of laser, Friedemann et al. [32] described the effect of CO_2_ laser stimulation at different acupoints on HR and mean arterial blood pressure in anesthetized rats. PC-6, ST-36, Quchi (LI-11), and Taichong (LR-3) were involved in the research. The results suggest that CO_2_ laser stimulation of all acupoints could regulate the cardiovascular functions in rats. Among them, laser stimulation at PC-6, ST-36, and LR-3 has the effect of increasing HR and mean arterial pressure, while stimulation at ST-36 has the opposite effect. A joint effort [33] from China and Austria initiated a study to investigate the therapeutic effects of invasive laser therapy, which are intravenous (i. v.) laser blood irradiation and interstitial (i. st.) LA at PC-6 on anesthetized rats. Physiological neurovegetative parameters such as HR and HRV and bioelectrical brain activity were analyzed in the study. The authors found that HR changed significantly only during i. st. LA stimulation, while during i. v. laser blood irradiation, they observed evidently increased LF/HF ratio of HRV and decreased integrated cortical electroencephalogram (EEG). Since the adequate laser dose has always been a controversial question in LA treatment, they conducted a further study [34] to find an optimal treatment time. They set up three different interstitial (i. st.) LA treatment duration groups and only found a significant reduction in HR during 20 min red laser stimulation, whereas no significant changes in 10 and 30 min stimulation appeared. In contrast to HR, the total HRV insignificantly increased during 20 min laser stimulation, and no significant difference was observed in the total HRV in any duration group. LA was reported to have the same function of regulating the autonomic nervous system as MA. A study conducted by Yang et al. [35] has investigated the effect of MA or LA at PC-6, ST-36, CV-12 (Zhongwan), and BL-21 (Weishu), respectively, on HRV and gastric motility in rats. The results proved that there was acupoint specificity in affecting gastric motility and HRV. MA or LA at PC-6 significantly increased gastric motility, HR, and LF/HF, stimulating ST-36 significantly enhanced gastric motility and HR, and CV-12 stimulation only significantly suppressed gastric motility, while BL-21 did not cause any parameters to change. The effect of MA or LA at PC-6 or ST-36 on gastric motility has been suggested to be associated with vagal activity.

### 3.3. HT-7

Shenmen (HT-7), located on the transverse crease of the wrist, is an important point in the heart meridian. HT-7 has been frequently used for treating neuropsychological impairments such as insomnia, amnesia, dementia, and anxiety neurosis [36–39]. Research studies [40] indicate that stimulating HT-7 has significant effects on brain activity. Thus, animal experiments are essential to investigate the molecular mechanism of LA at HT-7 on brain function. Sutalangka et al. [41] treated Alzheimer's disease rats with LA at HT-7. After treatment, the memory capacity of rats was improved. The underlying mechanisms were suggested to be related to the positive modulation effect of LA on oxidative stress and cholinergic function. LA at HT-7 has the ability to reduce oxidative stress and to enhance cognition. Wattanathorn and Sutalangka [42] focused on the treatment effect of LA at HT-7 on Parkinson's disease (PD). They found that neuron degeneration and cognitive impairment in PD rats could be improved by laser irradiation. Meanwhile, decreased oxidative stress and improved cholinergic and dopaminergic functions were considered as possible mechanisms. Oxidative stress plays a crucial role in autism pathophysiology. Although autism cannot be cured yet, LA is believed to be beneficial for the symptomatic treatment of autism. Khongrum and Wattanathorn [43, 44] conducted two animal experiments to elucidate the effect of LA at HT-7 on oxidative stress in the cerebrum and cerebellum of autism rats. The earlier study [43] reported that autism-like behavior in autistic rats was significantly improved by LA and accompanied by decreased oxidative stress in the cerebral cortex, striatum, and hippocampus. The latter study [44] focused on the cerebellar disorders' improvement. The results showed decreased oxidative stress, improved inflammation status, and enhanced GABAergic function in the cerebellar of autistic rats after LA treatment.

### 3.4. Other Single Points

There are fewer reports of animal experiments on LA at other single points. The conclusions drawn from these studies may be unconvincing due to the lack of repeated experimental studies, but they still made an indispensable contribution to the mechanism elucidation and widespread application of LA.

Baihui (GV-20) is one of the most commonly used points in acupuncture clinical practice. It is an acupoint of the Du meridian and is located on the highest place of the head. Baihui is a vital point for treating neurological and psychiatric diseases such as stroke, dizziness, headache, poor memory, and anxiety [45]. Animal experiments conducted by Jittiwat [46, 47] proved that LA at GV-20 could be beneficial for improving cognitive impairment and motor deficits in rats with cerebral ischemia by reducing the brain infarct volume, which may be related to antioxidant and anti-inflammatory effects of LA in the brain. Gao et al. [48] compared the effect of violet laser on different acupoints in anesthetized rats for autonomic nerve regulation. HR, HRV, and mean arterial blood pressure were measured. After irradiating GV-20, ST-36, and “heart” ear acupoint, respectively, only HR was significantly changed when stimulating GV-20.

Stimulating another Du meridian acupoint, which is called Mingmen (GV-2) on the waist with LA, has also been reported [49] to have anti-inflammatory and antioxidant effects. The recovery of the spinal cord in cats sustaining spinal cord injuries can be enhanced with yellow LA at GV-2 via the mitigation in apoptosis, oxidative stress, and inflammation in the lesion spinal cord.

Dubi (ST-35) is an acupoint located on the lateral knee eye, which belongs to the stomach meridian. It is a key point for knee disease. Zhao et al. [50] treated osteoarthritis mice with laser irradiation on ST-35, the result indicated that combined laser treatment of 650 nm and 10.6 *μ*m lasers significantly improved arthritic cartilage in mice, and the induction of heat shock protein (HSP) 70 in the arthritic chondrocytes was thought to be a potential mechanism for this effect. Wu et al. [51] further explored the therapeutic mechanism of laser moxibustion at ST-35 on monosodium iodoacetate (MIA)-induced knee osteoarthritis (KOA) in rats. They found that CO_2_ laser irradiation significantly inhibited the expression of inflammatory cytokines in the dorsal horn of the rat spinal cord. This result is consistent with their latest publication [52], which demonstrated that the analgesic effect of ST-35 laser moxibustion on KOA in rats could be exerted by inhibiting the neuroinflammation mediated by microglia activation. Pain relief has always been one of the main concerns in acupuncture research.

Taixi (KI-3), which is located in the ankle area, is an acupoint on the kidney meridian which plays a crucial role in curing kidney disease. The clinical application and mechanism of KI-3 have been widely researched. An animal experiment performed by Zeredo et al. [53] proved that infrared laser stimulation used alone on KI-3 or combined with conventional acupuncture needling has the effect of reducing pain in rats.

Kunlun (BL-60) is an acupoint on the bladder meridian, which is located at the ankle opposite Taixi. BL-60 is often used in combination with other points to treat stroke, lower back pain, and edema. Zhu et al. [54] observed the effect of low-power helium-neon laser at BL-60. Laser irradiation can reduce arthralgia and ankle swelling in rats with experimental arthritis.

The points of bladder meridian, which are located on the back, are called *back-shu* points. These points have effects of benefiting the internal organs. BL-23 (Shenshu) is one of the *back-shu* points which has a close relationship with kidney function. Zhang et al. [55] stimulate BL-23 in ovariectomized rats with three different doses of laser irradiation to explore the effect of LA on obesity in postmenopausal individuals. Beneficial effects were observed after treatment via reducing body weight and improving pituitary function. The dose of 30 J/cm^2^ was demonstrated to be the most effective dose among those applied (12, 30, and 60 J/cm^2^).

BL-20 (Pishu) is another *back-shu* point that benefits spleen function. Cornejo-Garrido et al. [56] demonstrated that LA with a wavelength of 650 nm and 980 nm, respectively, has a hypoglycemic effect on streptozotocin (STZ)-induced diabetic rats. Latief et al. [57] further proved the antihyperglycemic activity of LA at BL-20 in type 1 diabetic rats. The authors indicated that LA treatment could reduce fasting blood glucose (FBG) level, increase pancreatic beta-cell, and expand the Langerhans area.

### 3.5. LA on Multipoint

In clinical practice, the application of multipoint is more common than of single acupoint. Synergistic and complementary effects produced by the compatibility of different acupoints have greatly improved the clinical efficacy of acupuncture treatment. In 2001, a milestone of LA's technological development was the introduction of multichannel LA, which makes simultaneous stimulation of different acupuncture points possible (as with manual needle acupuncture in TCM) for the first time. Since then, more studies focusing on the mechanism of acupoint combinations have been reported in animal experiments of LA. The combination of two different acupoints is the most basic way of acupoint compatibility, and it is also the focus of basic research of LA on multipoint. Usually, these two acupoints have a certain correlation in TCM theory.

Sanyinjiao (SP-6) is a point on the spleen meridian. Spleen in TCM is associated with the stomach. Both are responsible for the absorption of food and liquids and transform them into nutrients available to the human body. SP-6 and ST-36 [58] are common matches for treating various gastrointestinal conditions. The combination of SP-6 and ST-36 has also been reported to have anti-inflammatory, analgesic, and blood circulation promoting effects in animal experiments. A study conducted by Marques et al. [59] evaluated the effect of SP-6 and ST-36 laser irradiation as an adjuvant for postoperative pain management in cats. The results indicated that although LA did not improve the pain scores, it significantly reduced the requirement of rescue medication. To further explore the analgesic effect of LA at SP-6 and ST-36, the same research group [60] compared the effects of LA and EA on rescue analgesia requirements in cats undergoing ovariohysterectomy. They found that preoperative EA and LA in combination with pain reliever significantly decreased the requirements for rescue analgesia. However, LA is more recommended for its painless, noninvasive, and easy-to-apply properties. The combination of SP-6 and ST-36 has also been involved in a study [61] of the effect of LA on longitudinal bone growth in adolescent rats. Low-level laser irradiation at SP-6 and ST-36 promoted longitudinal bone growth via significantly increasing bone growth rate and growth plate height. Handayani et al. [62] hypothesized that laser puncture affects serum concentration of insulin-like growth factor-1 (IGF-1) and ghrelin. They stimulated adolescent rats with LA on single or combined GV-20 and ST-36. The result indicated that IGF-1 affects the length of the lower limb, while ghrelin did not affect it. Laser irradiation on ST-36 and GV-20 + ST-36 was shown to increase serum concentrations of IGF-1. At the same time, another publication [63] from the same research group showed that the serum protein concentration was increased when stimulating ST-36 and GV-20 + ST-36 with LA in adolescent rats. In TCM theory, kidney strength is manifested in strong bones. Yongquan (KI-1) is an acupoint in the kidney meridian, which theoretically can be used to treat bone diseases. The combination of KI-1 and ST-36 was speculated to be efficient in preventing bone loss. Guo et al. [64] proved that laser irradiation on KI-1 and ST-36 could inhibit bone loss in rats caused by skeletal unloading via a systemic regulation. Besides, in their further investigation [65], they suggested that unloading-induced cartilage degeneration in rats can be reversed by the same laser therapy. KI-3 and BL-60 are located on either side of the Achilles tendon and are a common acupoint combination for the treatment of pain in the lower back, legs, and feet. Pan et al. [66] investigated for the first time the protective effects of LA at KI-3 and BL-60 on adjuvant-induced arthritis (AIA) rats. LA treatment was demonstrated that it significantly reduced ankle edema and hyperalgesia in AIA rats. It is suggested that LA attenuated cartilage degradation by reducing TNF-*α* activation and excavating extracellular matrix (ECM) macromolecules. In some studies LA has also been reported to have antiedema and antihyperalgesia effects [67]. Stimulating ST-36 and SJ-5 (Waiguan, an acupoint on Sanjiao meridian) with very low-intensity laser irradiation well controlled the pain and edema in acute inflammatory pain rats. Hegu (LI-4) is a point on the large intestine meridian. It is a key point for analgesia. LI-4 and ST-36 are frequently used in combination to treat painful diseases. By analyzing the records of tooth pulp generated somatosensory evoked potential (TPSEP), Sing and Yang [68] demonstrated a significant analgesic effect of laser stimulation on conscious rabbits at acupoints LI-4 and ST-36. Yun et al. [69] investigated the effect of LA on animal cognitive impairment. Transient focal cerebral ischemic rats were treated with LA or MA at the GV-20 and HT-7 point. The results suggested that LA treatment significantly improved rat memory and enhanced neuroprotective effect via regulating Creb, Bdnf, Bcl-2, and Bax gene expressions in the hippocampus. The effect and mechanism of laser acupoint irradiation on the brain tissue of rats with cerebral ischemia-reperfusion (CIR) were explored by Xiong and Li [70]. LA at GV-20, GV-2, and ST-36 has been proved to reduce CIR injury by improving energy metabolism, including enhancing the expression of GAP-43 and serum SOD and decreasing the serum MDA content. LA also reportedly affects white blood cell (WBC) count. The single and combined 10.6 *μ*m laser and 650 nm laser irradiation on ST-36 and GV-14 boosted the recovery of peripheral WBC counts in the rats with leucopenia [71]. Dazhui (GV-14) is an acupoint on Du meridian. It is a useful point for treating feverish disease. Another research [72] on this subject proves that, in addition to increasing the peripheral leukocyte count, the use of 10.6 *μ*m laser, 650 nm laser, and 10.6 *μ*m–650 nm compound laser upregulated the number of nucleated cells in the bone marrow, promoted cell cycle proliferation, reduced apoptosis, and improved the intramedullary hematopoietic system. Furthermore, in a recent study [73], LA was shown to regulate blood glucose. The combination of 10.6 *μ*m and 650 nm LA at BL-20, BL-23, and SP-6 had a positive effect on the regulation of hyperglycemia and insulin resistance in T2DM rats.

LA can be used in combination with other acupoint stimulation methods. In a case report conducted by Magden et al. [74], the authors treated a chimpanzee diagnosed with frequent ventricular premature complexes (VPCs) with combined MA and LA at PC-6 and HT-7. The report indicated that MA and LA therapy could decrease the mean number of VPC/min. Gong et al. [75] explored new methods to control the rise in blood pressure. They found that the treatment of electrical stimulation on ST-36, using low-level pulse laser irradiation on Erjian, and combination of laser irradiation and electrode stimulation on Erjian and ST-36 all have the therapeutic effect on improving hemorheology and blood pressure in spontaneously hypertensive rats. However, the combined acupoint stimulation method brought better results. In Table 1, the most important studies are documented.

## 4. Discussion

During the development of LA, continuous exploration and innovation of its technology have been observed, and the scope of its application is also expanding. Different from ordinary light sources, the laser has deeper penetration depth and generates particular energy, which can be used to stimulate acupuncture points as an alternative to a metal needle. It is crucial to pursue the curative effect without compromising safety. Thus, low-level laser stimulation, which is characterized by low output energy and no photothermal effect, is recommended in LA treatment. Studies [76, 77] have shown that there is a certain correlation between stimulus strength and stimulus effect in acupuncture. The Australian Medical Acupuncture College suggested [78] that the optimal energy density for LA and biological stimulation is 4 J/cm^2^. However, it is inconsistent with some studies [48], which support that the dose must be adjusted according to the individual responses. The basis of the dose-effect relationship comes from the specific effects of acupuncture. Yurtkuran et al. [79] investigated the effects and minimum effective dose of LA in knee osteoarthritis (KOA). Even patients have received very low-level laser irradiation with 10 mW/cm^2^ power density, 4 mW output power, 0.4 cm^2^ spot size, and 0.48 J dose per session, and LA was found to be effective in reducing periarticular swelling when compared with placebo laser. In classical acupuncture, acupoints located in different parts of the body are stimulated with metal needles of various sizes. In the process of LA, lasers of different colors are selected for treatment. One option is the green laser, for example, which is not as widely applied as red, near-infrared, or violet laser in the field of LA [76, 77]. Due to its superficial effect, it is often used for ear acupuncture therapy. Yellow laser is a promising option in the field of LA after the development of red, near-infrared, green, and violet lasers [80]. Although the penetration depth of the yellow laser is not as deep as red laser, the yellow laser can penetrate the human skull, which cannot be achieved by the red laser [81], and yellow laser stimulates the strongest natural photosensitizer, the hypericin from St. John's wort, which is a natural antidepressant that can be used in the treatment of depression. Therefore, yellow LA is maybe a promising approach for treating neuropsychiatric diseases. Acupuncture manipulation is one of the key factors affecting the effect of acupuncture in TCM [82]. The most commonly used manipulation methods in classical acupuncture include lift-thrust and twirl-twist. By changing the power of LA, manipulations can be achieved by laser needle [83–85]. In particular, the “lifting” operation is implemented by decreasing laser power and the “thrusting” operation by increasing laser power. The latest study [86] proved that LA with lift-thrust operation on PC-6 could raise the temperature of the fingertip more rapidly, stably, and longer-lasting than that without lift-thrust operation, while the same manipulation on a sham point nearby PC-6 (a point which is not conventionally defined as the acupoint) did not cause significant temperature changes. The realization of lift-thrust operation is extremely helpful in improving the performance of the LA and makes LA a promising alternative to MA [86]. Furthermore, compared to MA and EA, LA possesses more advantages in the basic research of acupuncture [87]. The radiation dose of LA can be varied with high accuracy, which makes it possible to quantify the stimulus strength of LA, thus ensuring the experiment has good repeatability. Furthermore, noninvasive, painless laser needle stimulation enables double-blind studies in the field of acupuncture research for the first time and causes less panic in animals, which is important for the treatment of emotional diseases. Therefore, in animal experiments, LA can not only replace MA but also has a broader range of applications than MA, and LA can alleviate the suffering of experimental animals. The use of LA for basic research of acupuncture should be encouraged.

However, some problems remain in current studies. Judging from the literature included in this study, the experimental animal research of laser acupuncture is still relatively limited. Only a small number of the acupuncture points are involved in the experimental animal research of LA, and there is no uniform standard for the laser parameter selection of LA. The stimulation intensity of LA treatment is also quite different. Moreover, studies have shown that in addition to traditional acupuncture, LA has a photobiomodulatory effect, but the research on how much the therapeutic effect of laser acupuncture depends on the property of acupoints is lacking. In the future, more acupuncture points should be involved in the animal experiment research of LA, and more attention should be paid to exploring the selection criteria of laser parameters in LA treatment and revealing the therapeutic effects of LA depending on the property of acupoints.

## Figures and Tables

**Table 1:** Characteristics of the involved studies.

References	Animal (*n*)	Acupoint/s	Laser parameters	Findings
Zhang et al. [29] 1986	Cats (57)	PC-6	Wavelength: 632.8 nm Output power: 3 mW	Low-power He-Ne laser irradiation can cause a relatively specific and immediate change in the electrocardiogram, mainly a change in HR, sometimes accompanied by an increase in R wave.
Bian et al. [16] 1989	Rabbits (25, *n* = 5 per group)	ST-36	Wavelength: 904 nm Output power: 3–5 mW	GaAs-laser irradiation can increase the pain threshold, and its analgesic effect is similar to that of needle acupuncture.
Zhu et al. [54] 1990	Rats (22, *n* = 8, 10 per group)	BL-60	Wavelength: 632.8 nm Output power: 2–3 mW	Low-power laser irradiation at local points can induce relief from arthralgia and reduction in the swelling of ankles, and it can also produce an instant analgesic effect in the test of average pain threshold.
Sing and Yang [68] 1997	Rabbits (12, *n* = 4 per group)	LI-4 and ST-36	Wavelength: 780 nm Output power: 5 mW	EA and LA have analgesic effects, as evidenced by consistently reducing the late near-field TPSEP peak-wave amplitude.
Zhao et al. [31] 2006	Rabbits (66, *n* = 11 per group)	PC-6	Wavelength: 0.65–0.66 *μ*m Output power: 36 mW Wavelength: 10.6 *μ*m Output power: 200 mW	Combined laser irradiation will certainly have a therapeutic effect on rabbits' bradycardia. A single CO_2_ laser can accelerate the recovery of bradycardia, but a single 650 nm laser irradiation does not.
Zeredo et al. [53] 2007	Rats (24, *n* = 8 per group)	KI-3	Wavelength: 2.94 *μ*m	High-intensity infrared laser stimulation may be used alternatively or in combination with conventional acupuncture needling for pain relief.
Cheng et al. [17] 2009	Rats (66, *n* = 11 per group)	ST-36	Wavelength: 0.65–0.66 *μ*m Output power: 36 mW Wavelength: 10.6 *μ*m Output power: 200 mW	Low-intensity compound 0.65–0.66 *μ*m laser and single 650 nm laser irradiation have a significant analgesic effect, and the mast cell rate positively related to its analgesic effect is considered to play an important role.
Yang et al. [22] 2010	Rats (40, *n* = 10 per group)	ST-36	Wavelength: 650 nm Output power: 10 mW	Both 650 nm laser and moxibustion pretreatment have an inhibitory effect on visceral traction pain, and its mechanism may be related to reducing the activity of AChE and the content of SP, and increasing the activity of LEK, and decreasing the expression of c-Fos protein and GFAP.
Lorenzini et al. [67] 2010	Rats (48)	ST-36 and SJ-5	Wavelength: 670 nm Output power: <0.03 mW	Acupoint stimulation using very low-intensity laser irradiation can control pain and edema in specific experimental conditions.
Guo et al. [64] 2010	Rats (18, *n* = 10 per group)	KI-1 and ST-36	Wavelength: 670 nm Output power: 5 mW	LA can inhibit bone loss in rats subjected to skeletal unloading via a systemic regulation.
Zhao et al. [50] 2011	Mice (60, *n* = 10 per group)	ST-35	Wavelength: 0.65–0.66 *μ*m Output power: 36 mW Wavelength: 10.6 *μ*m Output power: 200 mW	The combination of 650 nm and 10.6 *µ*m laser treatment resulted in significant improvement of arthritic cartilage in mice, which may be related to the induction of HSP70 in the arthritic chondrocytes.
Zhang et al. [55] 2011	Rats (70, *n* = 10 per group)	BL-23	Wavelength: 808 nm Output power: 100 mW	The semiconductor laser irradiation can result in photobiomodulation in OVX rats. The dose of 30 J/cm^2^ was the most effective dose among those applied (12, 30, and 60 J/cm^2^).
Wang et al. [65] 2012	Rats (18, *n* = 6 per group)	KI-1 and ST-36	Wavelength: 670 nm Output power: 5 mW	LA therapy can help maintain the quality of articular cartilage. For osteoarthritis caused by unloading.
Gong et al. [75] 2012	Rats (40, *n* = 10 per group)	Erjian and ST-36	Wavelength: 650 nm Output power: 50 mW	The combination of laser irradiation and electrode stimulation on Erjian and ST-36 all have the therapeutic effect on improving hemorheology and blood pressure in spontaneously hypertensive rats. However, the combined acupoint stimulation method brought better results.
Gao et al. [48] 2012	Rats (10)	GV-20, “heart“ ear point, and ST-36	Wavelength: 405 nm Output power: 1 mW	HR changes significantly during ultra-low-level violet laser stimulation of GV-20 in anesthetized rats. Total HRV changes insignificantly during violet laser application at GV-20, “heart” ear point, and ST-36. The LF/HF ratio of HRV showed no significant differences. Mean arterial pressure decreased after violet laser stimulation of GV-20 in rats.
Friedemann et al. and He et al. [32, 33] 2012	Rats (16)	PC-6, LI-11, ST-36, and LR-3	Wavelength: 10.6 *μ*m Output power: 0.5 W	Among the tested acupoints, LS stimulation only at ST-36 reduced HR and MAP. In addition, the effects were the most pronounced ones.
Erthal et al. [18] 2013	Rats (48, *n* = 8 per group)	ST-36	Wavelength: 830 nm Output power: 30 mW	LA was demonstrated to produce antinociceptive effects in rat models of peripheral inflammation. This effect is mediated by activation of the opioidergic and serotonergic (5-HT1 and 5-HT2A receptors) systems.
Yeom et al. [61] 2013	Rat (20, *n* = 10 per group)	ST-36 and SP-6	Wavelength: 635–680 nm Output power: 40 mW	LA promotes longitudinal bone growth in adolescent rats through the induction of BMP-2 and IGF-1.
Yang et al. [35] 2013	Rats (10)	PC-6 and ST-36	Wavelength: 658 nm Output power: 50 mW	The stimulatory effect of MA or LA at PC-6 or ST-36 on HR and gastric motility were associated with sympathetic nerve activity and vagal activity, respectively.
Sutalangka et al. [41] 2013	Rats (36, *n* = 6 per group)	HT-7	Wavelength: 405 nm Output power: 100 mW	LA has a positive modulation effect on cholinergic function, which in turn leads to reduced cognitive impairment in an animal model of Alzheimer's disease.
He et al. [33] 2013	Rats (10 in one group)	PC-6	Wavelength: 658 nm Output power: 50 mW	HR changed significantly during i. st. LA stimulation in anesthetized rats. Total HRV increased insignificantly during i. v. and i. st. laser stimulation. The LF/HF ratio of HRV showed significant changes only during i. v. laser blood irradiation. Integrated cortical EEG decreased insignificantly during EA and i. v. laser blood irradiation.
He et al. [34] 2013	Rats (6)	PC-6	Wavelength: 658 nm Output power: 50 mW	Different treatment times led to different effects on neurovegetative and neurobioelectrical parameters. Significant changes in HR and total HRV were only observed at 20 minutes of laser stimulation compared to 10 and 30 minutes of stimulation.
Zhao [71] 2014	Rats (66, *n* = 11 per group)	ST-36 and GV-14	Wavelength: 0.65–0.66 *μ*m Output power: 36 mW Wavelength: 10.6 *μ*m Output power: 200 mW	The single and combined 10.6 *μ*m and 650 nm laser irradiation on ST-36 and GV-14 accelerated the recovery of the WBC count in the rats with leucopenia.
Wattanathorn and Sutalangka [42] 2014	Rats (48, *n* = 12 per group)	HT-7	Wavelength: 405 nm Output power: 100 mW	LA can improve neuron degeneration and memory impairment in an animal model of PD9 partly via the decreased oxidative stress and the improved cholinergic and dopaminergic functions.
Marques et al. [59] 2015	Cats (20, *n* = 10 per group)	SP-6 and ST-36	Wavelength: 904 nm	LA reduced postoperative analgesic requirements in cats undergoing ovariohysterectomy.
Khongrum and Wattanathorn [43] 2015	Rats (40, *n* = 10 per group)	HT-7	Wavelength: 405 nm Output power: 100 mW	LA partly mitigated autistic-like symptoms via improved oxidative status.
Erthal and Nohama [19] 2015	Mice (32, *n* = 8 per group)	ST-36	Wavelength: 830 nm Output power: 30 mW	Photonic stimulus with LLLT produces an antinociceptive effect in chronic models of nociception and inflammation.
Magden et al. [74] 2016	Chimpanzee (1)	PC-6 and HT-7	—	Acupuncture and laser therapy appeared to decrease the mean number of VPC/min in this chimpanzee.
Frederico et al. [23] 2016	Rats (10, *n* = 5 per group)	ST-36	Wavelength: 904 nm Output power: 25 mW	Stimulation of ST-36 does lead to biological phenomena that interfere with the metabolism of the thyroid.
Erthal et al. [20] 2016	Mice (40, *n* = 8 per group))	ST-36	Wavelength: 830 nm Output power: 30 mW	LLLT produced a relevant anti-inflammatory effect and reduced edema, temperature, and free radicals' levels in mice paw.
Liu et al. [25] 2017	Mice (18, *n* = 6 per group)	ST-36	Wavelength: 633 nm Output power: 10 mW	Fiber LA improved the tolerance to hypoxia, reduced the HR, and enhanced the expression of serum IL-1 level.
Yun et al. [69] 2017	Rats (24, *n* = 6 per group)	GV-20 and HT-7	Wavelength: 650 nm Output power: 30 mW	LA treatment could improve cognitive impairment in MCAO rats to enhance the cholinergic system in the hippocampal CA1 region and to exert a neuroprotective effect by regulating Creb, Bdnf, Bcl-2, and Bax gene expressions.
Khongrum and Wattanathorn [44] 2017	Rats (48, *n* = 12 per group)	HT-7	Wavelength: 405 nm Output power: 100 mW	LA decreased oxidative stress status, inflammation, and improved GABAergic function, which make it a potential strategy to improve the cerebellar disorders in the VPA-rat model of autism.
Zhang et al. [24] 2018	Mice (18, *n* = 6 per group)	ST-36	Wavelength: 633 nm Output power: 10 mW	Both hollow needle and optical fiber needle reduced the concentration level of porphyrin, NADH, lipid, and glucose but elevated the concentration level of phosphatidylinositol in blood.
Zeng et al. [21] 2018	Rats (36, *n* = 5–7 per group)	ST-36	Wavelength: 650 nm Wavelength: 830 nm	Repetitive LLLA treatments ameliorated PI-induced mechanical pain, which may be related to the inhibition of PI-induced p-ERK, p-p38, and iNOS.
On-Ong-Arj et al. [49] 2018	Rats	GV-2	Wavelength: 589 nm Output power: 50 mW	Yellow laser enhanced the recovery of the spinal cord via the increase in BDNF and the decrease in inflammation, apoptosis, and oxidative stress status in the lesion spinal cord.
Handayani et al. [62] 2018	Rats (40, *n* = 20 per group)	GV-20 and ST-36	Wavelength: 635–680 nm Output power: 5 mW	LA at ST-36 has the most significant effect on increasing protein concentration in rats, although the difference was not statistically significant.
Handayani et al. [63] 2018	Rats (40, *n* = 20 per group)	DU-20 and ST-36	Wavelength: 635–680 nm Output power: 5 mW	LA administered to both the ST-36 and GV-20 + ST-36 subgroups increased serum concentrations of IGF-1 in adolescent rats.
Pan et al. [66] 2019	Rats (30, *n* = 15 per group)	BL-60 and KI-3	Wavelength: 780 nm Output power: 50 mW	LA attenuated cartilage degradation in rats with AIA-induced cartilage damage by suppressing TNF-alpha activation and upregulating ECM macromolecules.
Nascimento et al. [60] 2019	Cats (30, *n* = 11 per group)	ST-36 and SP-6	Wavelength: 904 nm Output power: 70 mW	Both EA and LA, in combination with a single dose of tramadol, reduced the postoperative analgesic requirements following OHE in cats.
Liu et al. [72] 2019	Rats (66, *n* = 11 per group)	GV-14 and ST-36	Wavelength: 10.6 *μ*m Output power: 80 mW Wavelength: 650 nm Output power: 36 mW	LA treatment increased the number of nucleated cells in the bone marrow, decreased the unfavorable effects of cyclophosphamide on the cell cycle, induced the cell cycle towards proliferation, decreased apoptosis, improved the intramedullary hematopoietic system, and increased peripheral leukocyte count.
Jittiwat [47] 2019	Rats (40, *n* = 10 per group)	GV-20	Wavelength: 810 nm Output power: 100 mW	LA alleviated cognitive impairment and motor deficits via antioxidant and anti-inflammatory effects in focal ischemic rats.
Li et al. [52] 2020	Rats (24, *n* = 8 per group)	ST-35	Wavelength 10.6 *μ*m Output power 80 mW	Laser moxibustion significantly alleviated MIA-induced KOA pain through inhibition of the microglial activation-mediated neuroinflammation.
Li et al. [73] 2020	Rats (40, *n* = 10 per group)	BL-20, BL-23, and SP-6	Wavelength: 10.6 *μ*m Output power: 80 mW	The compound LA-moxibustion of 10.6 *µ*m and 650 nm had positive effects on the regulation of hyperglycemia and insulin resistance in T2DM rats.

He-Ne = helium-neon; AlGaAs = aluminum gallium arsenide; GaAs = gallium arsenide; LA = laser acupuncture; EA = electroacupuncture; MA = manual acupuncture; TPSEP = tooth pulp generated somatosensory evoked potential; OVX = ovariectomized; LS = infrared laser stimulation; MAP = mean arterial blood pressure; HR = heart rate; HRV = heart rate variability; LF/HF = high frequency/low frequency; BMP-2 = bone morphogenetic protein-2; IGF-1 = insulin-like growth factor-1; i. v. = intravenous; i. st. = interstitial; PD = Parkinson's disease; LLLT = low-level laser treatment; VPC = ventricular premature complexes; MCAO = middle cerebral artery occlusion; GABA = gamma-aminobutyric acid; VPA = valproic acid; NADH = nicotinamide adenine dinucleotide; PI = plantar incision; p-ERK = protein kinase R (PKR)-like endoplasmic reticulum kinase; p-p38 = phosphorylated protein-38; iNOS = inducible nitric oxide synthase; BDNF = brain-derived neurotrophic factor; IGF-1 = insulin-like growth factor-1; AIA = adjuvant-induced arthritis; ECM = extracellular matrix; TNF-alpha = tumor necrosis factor-alpha; OHE = ovariohysterectomy; MIA = monosodium iodoacetate; KOA = knee osteoarthritis; 5-HT = serotonin.

## Data Availability

The original data and references used to support the findings of this study are available from the first and the corresponding author upon request.

## Abbreviations

AIA: Adjuvant-induced arthritis
AlGaAs: Aluminum gallium arsenide
BDNF: Brain-derived neurotrophic factor
BMP-2: Bone morphogenetic protein-2
CFA: Complete Freund's Adjuvant
CIR: Cerebral ischemia-reperfusion
EA: Electroacupuncture
ECM: Extracellular matrix
FBG: Fasting blood glucose
GaAs: Gallium arsenide
GABA: Gamma-aminobutyric acid
GFAP: Glial fibrillary acidic protein
He-Ne: Helium-neon
HR: Heart rate
HRV: Heart rate variability
IGF-1: Insulin-like growth factor-1
i. st.: Interstitial
iNOS: Inducible nitric oxide synthase
i. v.: Intravenous
KOA: Knee osteoarthritis
LA: Laser acupuncture
LEK: Leu-enkephalin
LF/HF: High frequency/low frequency
LLL: Low-level laser
LLLA: Low-level laser acupuncture
LLLT: Low-level laser treatment
LS: Infrared laser stimulation
MA: Manual acupuncture
MAP: Mean arterial blood pressure
MCAO: Middle cerebral artery occlusion
MIA: Monosodium iodoacetate
NADH: Nicotinamide adenine dinucleotide
OHE: Ovariohysterectomy
OVX: Ovariectomized
PBM: Photobiomodulation
PD: Parkinson's disease
p-ERK: Protein kinase R (PKR)-like endoplasmic reticulum kinase
PI: Plantar incision
p-p38: Phosphorylated protein-38
PSNL: Partial sciatic nerve ligation
5-HT: Serotonin
STZ: Streptozotocin
TCM: Traditional Chinese medicine
TPSEP: Tooth pulp generated somatosensory evoked potential
TNF-alpha: Tumor necrosis factor-alpha
VPA: Valproic acid
VPC: Ventricular Premature Complexes
WBC: White blood cell.
